# Searching for Possible Links between Alzheimer’s Disease and Systemic Sclerosis

**DOI:** 10.31138/mjr.31.4.378

**Published:** 2020-12-28

**Authors:** Efthymios Dardiotis, Dimitrios P. Bogdanos

**Affiliations:** 1 Department of Neurology and Clinical Immunology, Faculty of Medicine, School of Health Sciences, University of Thessaly, University General Hospital of Larissa, Larissa, Greece; 2 Department of Rheumatology and Clinical Immunology, Faculty of Medicine, School of Health Sciences, University of Thessaly, University General Hospital of Larissa, Larissa, Greece

**Keywords:** Autoimmunity, immunology, neurogeneration, pathogenesis, scleroderma

Alzheimer’s disease (AD) is a chronic neurodegenerative disorder characterised progressive cognitive and functional decline. The prevalence of AD ranges from 4.3% to 11.3% in European elderly populations over 65 years of age.^1,2^ Although degenerative processes in the blood-barrier protected brain neurons have long be considered as dominant in the pathophysiology of AD, mounting evidence especially from genetic studies including ours,^3–5^ and functional studies have identified immune system cells as critical players in initiation, maintenance or resolution of these processes.

Innate immunity appears pivotal for the homeostasis of central nervous system and imbalance of the immune system participates in the induction and progression of neurodegenerative diseases such as Alzheimer's disease, Parkinson's disease, Huntington's disease, amyotrophic lateral sclerosis, and frontotemporal dementia. Such diseases are characterised by a dysregulated immune system as demonstrated by the abnormal activation of innate immune cells, microglia, astrocytes, mast cells, and macrophages, which are participating in the inflammatory and degenerative processes, inflicting damage to the central nervous system.^6^

Dysregulation of brain innate immune cells, such as resident microglia, and lack of regulated innate and adaptive immunity, that can influence microglial phenotype, emerged as key elements in AD pathogenesis, but at the same time, represent potential targets for therapeutic interventions.^7^

Over the last 20 years, the theoretical background for an «autoimmune cause» participating as a trigger factor for the development of AD has gained traction,^8–10^ mainly due to the appreciation that, in the pathogenesis of the disease, an important role is played by the blood brain barrier, neurons, astrocytes, microglia, lymphocytes, CD14+ monocytes, macrophages, and a plethora of cytokines such as IL-6, IL-1 and IL-10,^11,12^ and the fact that adaptive immune responses leading to neuronal degeneration may indeed exist in animal models, as well as in humans.^13–16^ Interestingly, combined brain-heart magnetic resonance imaging studies in patients with autoimmune rheumatic diseases and concomitant cardiovascular diseases have more frequently specific brain lesions compared to age- and sex-matched individuals with cardiovascular disease, further pointing towards an instrumental role of autoimmunity, lined to connective tissue disorders, in neuropathological manifestations.^17^ Such imaging approaches may indeed assist efforts to search in depth the link between brain, heart, and autoimmune rheumatic diseases.

However, T cell responses to neural autoantigens appears comparable in AD patients and age-matched healthy controls,^18^ raising concerns as to whether antigen-specific, adaptive immunity is participating in the development of the disease, and raising doubts as to whether the pathogenesis of the disease has indeed immunological origins.^18^

On the other hand, a hypothesis has been formulated suggesting that breaking peripheral immunological tolerance to central nervous system self-antigens and initiation of “protective autoimmunity” may indeed help fighting acute and chronic neurodegenerative conditions, such as AD.^19^ On that basis, it is difficult to understand whether autoimmune phenomena play a role in the induction and/or progression of AD. Epidemiological studies assessing the co-occurrence of AD and autoimmune diseases could be of help in addressing this matter. Thus, studies which are focused on assessing patients with both AD and autoimmune diseases (which by *de novo* are the final outcome of a dysregulated immune system) are of great importance, as they could provide the impetus for aetiopathological connotations in case of documented relation between AD and a given autoimmune disorder. Such studies may assist efforts to understand the role of the immune system in neurodegenerative processes and help us delineate the presumed pathogenic link between AD and autoimmune diseases in general. As autoimmune diseases are heterogenous, largely divided into organ-specific and no-organ specific, it would be of interest to learn whether AD is associated with a particular disease or not.

Neurodegeneration in AD is triggered by the presence of misfolded deposits of proteins such as Amyloid β (Aβ) and tau. Aβ peptides are produced from amyloid precursor protein (APP) inside neurons when specific cleaving enzymes are activated via the amyloidogenic pathway. Aβ is then secreted into the extracellular space where it forms neurotoxic aggregates (soluble oligomers, insoluble polymers).^20^ In parallel with Aβ, intracellular hyperphosphorylated tau species have toxic effects by aggregating into the neurofibrillary tangles or by impairing the stability of microtubules and compromising mitochondrial function.^20^ Aβ and tau aggregates affect the structure and function of neurons leading to reduced number of neurons or synapses, loss of synaptic vesicles, damaged neurotransmitter receptor, and impaired synaptic plasticity. These changes to neurons are associated with cognitive decline in AD.^21^

The other side of the coin is chronic brain neuroinflammation in AD brains. It is mainly triggered by innate immune response, and it seems to be an integral part of AD pathophysiology pathogenesis and progression. Microglia are activated and migrated by the presence of ab species or the production of complement components and clear Aβ via phagocytosis. Phagocytosis of Aβ, apart from brain-resident microglia, is also performed by blood-derived myeloid cell infiltration from the periphery. Activation of adaptive immunity during the beginning or the course of the disease may have regulatory effects on the infiltration of peripheral cells into the CNS or on influencing the microglial phenotypes.^22^ However, innate and adaptive immune responses in AD are impaired.^23,24^ Microglia are dysfunctional in AD, either via aberrant activation, which can be absent or exaggerated, and/or through decreased phagocytic capacity.^25^ This may lead to a vicious circle between neurodegeneration and neuroinflammation, the immune system playing a crucial role in this close, never-ending interplay.

What is very promising for future interventions is that microglial activation in AD patients can be visualised in positron emission tomography (PET) scan using a biomarker that is expressed in activated microglia. In a recent, interesting longitudinal PET study in mild cognitive impairment (MCI) and AD patients, microglial activation followed a dual peak course with an early reduction in MCI and a late wave of activation in AD.^26^ The first peak may represent an initial effort of microglia to clear Ab toxic deposits by adopting a neuroprotective phenotype, which, as the amyloid clearance fails, are turned into a toxic pro-inflammatory phenotype that will inevitably lead to “immunological paralysis” of microglial cells.^23^

Microglial activation and switch between different phenotypes may be influenced by systemic inflammation and various peripheral conditions such as trauma, obesity, metabolic syndrome, insulin resistance, and chronic periodontitis.^27^ Furthermore, a number of systemic abnormalities such as cardiovascular diseases, hepatic or renal disfunction, respiratory and sleep disorders, blood abnormalities, and metabolic disorders were found to deteriorate the course of AD. In addition, microbiota disturbances or infections as well as systemic inflammation and disorders of systemic immunity are critical co-morbidities that exacerbate AD.^28^ Thus, optimal management of comorbidities is crucial in the course of AD and vice versa.

A recent study by Watad et al.^29^ linked AD with systemic sclerosis (SSc), and offer offered valuable clues in understanding the complex interplay between neurodegeneration, neuroinflammation, and autoimmunity. SSc is a chronic, rare, autoimmune rheumatic disease characterised by small vessel vasculopathy and progressive fibrosis of the skin and internal organs.^30^ Neurological involvement in SSc patients is not expected to be very frequent.^31^ Such an involvement includes seizures, headache, cranial nerve involvement, hemiparesis, depression and anxiety, myopathy, carpal tunnel syndrome, peripheral sensorimotor polyneuropathy, and autonomic neuropathy. Cognitive impairment was detected in approximately 8.5% of SSc patients.^31^ However, asymptomatic white matter lesions were found in an increasing number of studies in SSc patients. Given the dysregulation of immune responses in both SSc and AD, the question is whether there is any epidemiologic, clinical, or pathogenetic link between the two diseases.

Watad et al.,^29^ in their work, examined the potential link between SSc and AD and the effect of both diagnoses on the patients’ survival. They performed a large population-based case-control study using data extracted from the largest healthcare registry in Israel, the Clalit Health Services, which covers almost half of the Israeli population. In this Real-World Data approach, that eliminates specialist centres referral bias, a relatively large cohort of 2,431 SSc patients were compared with 12,377 controls matched for age and gender.

Several important findings were drawn from this comparison. At first, the frequency of AD did not differ between SSc patients (5.5%) and controls (5.9%). On multivariate logistic regression analysis, adjusting for possible AD confounders, SSc was not found to be an independent risk factor for AD. This finding does not seem to be unexpected and it comes to confirm that SSc pathophysiology does not trigger αβ-peptide accumulation processes, leading to neuronal degeneration and AD.

Regarding mortality rates, the cox multivariate survival analysis, after adjusting for possible risk factors, showed that the diagnosis of SSc was associated with an increased risk of all-cause mortality by a Hazard ratio (HR) of 2.35 (95% CI: 2.05 – 2.69). Similarly, AD was an independent predictor of death (HR: 2.19, 95% CI: 1.94 – 2.48).

Importantly, among AD patients, as shown by Kaplan-Meier survival analysis without correcting for potential confounders, the diagnosis of SSc did not significantly affect survival (HR: 1.13, 95% CI: 0.68 – 1.89). On the contrary, among SSc patients the diagnosis of AD significantly decreased survival by an HR of 2.35 (95% CI: 1.44 – 3.83).

In a nutshell, the study by Watad et al.^29^ stresses the importance of SSc and AD as co-morbidities on the patients’ survival, whereas the novel finding is the unfavourable effect of AD on SSc outcome. These findings warrant further investigation, especially whether these results are still present in the various clinical variants of SSc, and importantly, whether early screening and detection of AD, appropriate, and vigorous pharmacological and non-pharmacological interventions of AD in patients with SSc may lead to better clinical course and increased survival.
